# Insights into the molecular mechanisms of browning tolerance in luffa: a transcriptome and metabolome analysis

**DOI:** 10.3389/fpls.2025.1530531

**Published:** 2025-06-10

**Authors:** Shan Wu, Miao Sun, Huashan Lian

**Affiliations:** School of Landscape Architecture, Chengdu Agricultural College, Wenjiang, China

**Keywords:** browning, transcriptome, metabolome, flavonoid metabolism, antioxidant enzyme, transcription factor

## Abstract

**Introduction:**

Enzymatic browning significantly affects the edible, nutritional, and commercial value of luffa. Investigating the expression and regulation of key enzyme genes involved in the browning process is crucial for understanding the molecular mechanisms underlying luffa browning.

**Methods:**

Fruit samples were collected at 15 (S1), 20 (S2), and 45 days (S3) after flowering from two contrasting luffa varieties: the browning-sensitive Long-quan-yi (LQY) and the browning-tolerant Jiang-du (JD). RNA-sequencing technology, combined with ultra-performance liquid chromatography electrospray ionization tandem mass spectrometry (UPLC-ESI-MS/MS), was used to obtain transcriptome and metabolome data, which were subsequently analyzed using a series of bioinformatics approaches. Quantitative polymerase chain reaction (q-PCR) was used to validate gene expression.

**Results:**

Compared with JD, the ROS levels and PPO activity were elevated in LQY. In the polyphenol metabolic pathway, 24 key enzyme genes including *CuAO*, *PPO*, and *TDC*, were identified. In the flavonoid metabolic pathway, 57 key structural genes, such as *PAL*, *C3H*, and *4CL*, were identified. These genes showed different expression patterns between the two luffa varieties. Differentially expressed genes were mainly involved in the regulation of 34 MYB, 15 bHLH, 19 WD40, and 14 WRKY transcription factors. Further metabolomics analysis showed that the levels of polyphenol metabolites were upregulated in LQY, whereas the levels of flavonoid metabolites were upregulated in JD.

**Discussion:**

This study integrated transcriptomic and metabolomics data to identify key genes, transcription factors and metabolic pathways associated with luffa browning. q-PCR analysis was performed to validate the expression of *POD* and *MYB* genes. These findings provide a theoretical foundation for further investigation into the molecular mechanisms underlying luffa browning and offer potential targets for genetic improvement or breeding strategies to enhance luffa quality.

## 1 Introduction

Sponge gourd [*Luffa cylindrica* (L.) Roem (*L. cylindrica*), syn. *L. aegyptiaca* Mill] is an annual melon vegetable crop belonging to the family Cucurbitaceae. Most luffa varieties are susceptible to browning in the peel, pulp and soup during storage, transportation and cooking, which significantly affects their edibility, nutritional quality, and commercial value (Bustos et al., 2015). Understanding the molecular mechanisms underlying luffa browning and developing strategies to mitigate browning have become key objectives in luffa breeding.

Browning is a common phenomenon in the issues of horticultural crop. Based on underlying mechanisms, browning can be classified into two categories: enzymatic browning, which involves the oxidation of phenolic compounds by oxidases to form quinones, and non-enzymatic browning, which occurs without oxidase participation (Du et al., 2012). The browning of luffa belongs to enzymatic browning. This process is a physiological and biochemical response to stress, which triggers an increase in reactive oxygen species (ROS), damages the structure of cells, and then breaks the compartmentalized separation of phenolic compounds and oxygen. Under the catalysis of polyphenol oxidases (PPOs) and peroxidases (PODs), phenolic substances in the tissue are oxidized, leading to browning (Khajuria et al., 2007; Lante et al., 2016). Previous research on luffa browning focuses on the enzymatic properties, activity inhibition, browning extent and total phenol content during enzymatic browning. The 1% CaCl2-treated luffa exhibited higher activities of superoxide dismutase (SOD), catalase (CAT), and phenylalanine ammonia lyase (PAL), along with lower PPO activity, compared with the untreated luffa (Feng et al., 2022). Chang et al. analyzed PPO and POD activities during fruit growth and development across three luffa cultivars, and observed cultivar- and tissue-specific variations (Maohung et al., 2010). These studies demonstrate a direct correlation between the content and activity of PPO and POD with the extent of browning, further supporting the notion that luffa browning is primarily enzymatic.

As omics technology advances, researchers have increasingly focused on elucidating the molecular mechanisms underlying luffa browning. Transcriptomic analyses have facilitated the identification and cloning of PPO, POD and other oxidase genes involved in this process (Wang et al., 2020; Xu et al., 2017). Chen et al. investigated the transcriptome profiles of towel gourd varieties with differing browning sensitivities, revealing that browning may be linked to signal transduction pathways associated with phenolic oxidation, as well as carbohydrate and hormone metabolism (Chen et al., 2015). Wang et al. used high-throughput sequencing to analyze the transcriptomes of the browning-tolerant variety ‘ 2d-2 ‘ and the browning-prone sensitive ‘ 35d-7 ‘, revealing that differences in the expression of enzymatic reaction-related genes (*PPO*, *POD*) and certain transcription factors (TFs) (such as the WRKY family) may contribute to variations in browning tolerance (Wang et al., 2020). Zhu et al. employed RNA-sequencing (RNA-seq) to investigate transcriptomic changes in the luffa variety ‘Fusi-3,’ identifying 11 genes from five gene families (*PPO*, *PAL*, *POD*, *CAT*, and *SOD*) and four WRKY TF-related genes associated with enzymatic browning (Zhu et al., 2017). Wang et al. conducted a metabolomic analysis of the browning-sensitive luffa variety ‘35D-7’ using ultra-performance liquid chromatography-mass spectrometry (UPLC-MS), and identified coniferaldehyde, syringin, and isochlorogenic acid A as key phenolic acid metabolites involved in the browning process (Wang et al., 2021). Wen et al. applied ultra-high-performance liquid chromatography (UHPLC) to characterize phenolic compounds in luffa, identifying gentisic acid as the predominant phenolic compound (Wen et al., 2016). Current research on the molecular genetic mechanisms behind luffa browning tolerance remains limited. During our breeding processes of luffa varieties, two contrasting materials stood out: Long-quan-yi (LQY), which was sensitive to browning, and Jiang-du (JD), which demonstrated tolerance to browning. These two varieties were therefore selected in this study to explore the underlying mechanisms of browning tolerance. Additionally, RNA-seq and metabolomic techniques were used to identify differentially expressed genes (DEGs) and differential metabolites (DAMs) in luffa fruit at 15 days (S1), 20 days (S2) and 45 days (S3) following flowering. This study provides new insights into the molecular and metabolic mechanisms of luffa browning, offering a reference for the utilization of browning-tolerant materials and the improvement of luffa varieties.

## 2 Materials and methods

### 2.1 Plant materials

Two luffa varieties were used in this study: browning-sensitive LQY and browning-tolerant JD. All luffa plants were cultivated in the experimental field of Chengdu Agricultural College, China. Luffa fruits were harvested at 15, 20 and 45 days after flowering (April to May 2021), designated as S1, S2 and S3, respectively. These stages were selected based on the life cycle of luffa to capture the onset, progression, and culmination of physiological and biochemical changes potentially associated with fruit browning. At the same time, flowers, leaves and stems were collected at the corresponding time points. The samples were frozen immediately in liquid nitrogen, followed by separation of the seeds and receptacles under frozen conditions.

### 2.2 Morphological examination of luffa fruit slices

Fresh luffa fruits were collected 45 days after flowering, and then manually sliced through the middle. The slices were left at room temperature for 15 minutes to observe browning in the cross-section.

### 2.3 Measurements of PPO activity and ROS levels

PPO activity was measured using spectrophotometry, following the method of González et al. (2020). Specifically, 1 g of frozen sample was homogenized in 5 mL of cold PPO extraction buffer. The homogenate was transferred to a 2-mL centrifuge tube and centrifuged at 8,000 rpm at 4°C for 10 min. Next, 150 µL of the supernatant was mixed with 750 µL of PPO assay buffer (50 mM sodium phosphate buffer, pH 6.0; 0.1% SDS; 15 mM 4-methylcatechol) in a new tube. The mixture was incubated at 37°C for 10 min, and immediately transferred to a 97°C boiling water bath for 5 min. After centrifugation at 10,000 rpm for 10 min at room temperature, the supernatant was transferred to a quartz cuvette, and absorbance was measured at 420 nm (A420 nm). PPO enzymatic activity was defined as one unit (1 U), representing the amount of enzyme required to increase absorbance at 420 nm by 0.01 per min in 1 g of tissue within a 1-mL reaction system.

ROS level measurement was performed as previously described with slight modifications (AbdElgawad et al., 2016). Frozen samples were ground in liquid nitrogen, and 1 g of the resulting powder was added to 9 mL of sodium phosphate buffer (100 mM, pH 7.0). The mixture was vortexed at 3500 rpm, and centrifuged for 10 min to obtain the supernatant as a 10% homogenate. Subsequently, 20 µL of the supernatant and 20 µL of the enzyme solution were added to 200 µL of substrate application solution. The mixture was incubated at 37°C for 20 min. Absorbance was then measured at 450 nm using a microplate reader (MK3; Thermo Fisher Scientific, Waltham, MA, USA). ROS concentration was calculated as: c=A/(ε×b) where ‘c’ represents the sample concentration, A is the absorbance value, ϵ is the molar extinction coefficient, and b refers to the path length of the sample. PPO and ROS measurements were conducted in triplicate, including three biological and technical replicates.

### 2.4 Transcriptome sequencing and analysis

Total RNA was extracted from each frozen fruit sample using an RNA prep Pure Plant Plus Kit (Tiangen, Beijing, China), and mRNA was obtained using oligo (dT) beads (Beyotime, Shanghai, China). The mRNA was reverse transcribed to cDNA, and the library was constructed using an NEBNext^®^ Ultra™ RNA Library Prep Kit for Illumina^®^ (NEB, Ipswich, MA, USA) according to manufacturer’s instructions. Three biological replicates were included to ensure the reliability of transcriptome data. Qualified libraries were paired-end sequenced on the Illumina HiSeq 6000 sequencing platform at Yunnan Pulis Biotechnology Co. Ltd. (Kunmin, China), yielding over 6 GB of raw data per sample.

Clean reads obtained after filtering the raw data were aligned to the luffa reference genome (https://www.ncbi.nlm.nih.gov/, PRJNA596077) using HISAT2 (v2.1.0) (Kim et al., 2019). The number of reads that mapped to each gene was quantified using HTSeq (v0.6.1) (Anders et al., 2015). The fragments per kilobase of transcript sequence per million base pairs sequenced (FPKM) of each gene were then calculated based on gene length and the number of mapped reads. Principal component analysis (PCA) was performed using the prcomp function (Maćkiewicz and Ratajczak, 1993) in R to compute the variance ratio explained by each principal component (PC). DEGs were identified using the DESeq2 package (v4.0.4) (Love et al., 2014) in R. P-values were adjusted using the Benjamini-Hochberg procedure to control the false discovery rate (FDR). DEGs were considered significant with P-adjusted value < 0.05 and |log2(fold change)| > 1. Functional enrichment analysis of the DEGs was performed using the clusterProfiler package (Yu et al., 2012) in R, with the KEGG database as reference.

### 2.5 Metabolite extraction and ultra-performance liquid chromatography electrospray ionization tandem mass spectrometry analysis

Freeze-dried fruit material was crushed for 1.5 min at 30 Hz using a mixer mill (MM 400, Retsch, Haan, Germany) with zirconia beads. A total of 100 mg of the powder was extracted overnight with 1.2 mL of 70% aqueous methanol at 4°C. The extracts were then filtered (SCAA-104, 0.22 μm pore size; ANPEL, Shanghai, China) after centrifugation at 12000 rpm for 10 min. Three biological replicates were included.

The samples were examined using a UPLC–ESI–MS/MS system (UPLC, Nexera X2, SHIMADZU, Japan; MS, 4500 Q TRAP, Applied Biosystems, USA). The UPLC system used an Agilent SB-C18 column (1.8 µm, 2.1 mm*100 mm) (Agilent, Santa Clara, CA, USA). The mobile phase consisted of solvent A (0.1% formic acid in water), and solvent B (0.1% formic acid in acetonitrile). A gradient program was adopted, starting with 95% A and 5% B. A linear gradient was applied over 9 min, gradually reaching 5% A and 95% B, which was maintained for 1 min. The composition was adjusted back to 95% A and 5% B within 1.1 min and held for 2.9 min. The column oven was maintained at 40°C, and the injection volume was 4 µL. The effluent was then introduced into an ESI-triple quadrupole-linear ion trap (QTRAP)-MS. PCA was performed using the prcomp function in R, with the data subjected to unit variance scaling prior to analysis. Metabolites with VIP values ≥ 1 and absolute Log2 fold changes (|Log2FC|) ≥ 1 were considered significantly regulated across groups. The annotated metabolites were linked to the KEGG database. Pathways with substantially upregulated metabolites were then loaded into metabolite set enrichment analysis (MSEA), and their significances were established using the p-values of the hypergeometric test.

### 2.6 Correlation analysis between DEGs and DAMs

Pearson correlation coefficients between DEGs and DAMs were calculated using the cor function in R. DEGs and DAMs with correlation coefficients greater than 0.80 and p-values less than 0.05 were selected for further analysis. Enrichment analysis of the significantly correlated DEGs and DAMs was subsequently conducted using the KEGG database to identify associated biological pathways.

### 2.7 Quantitative real–time polymerase chain reaction (q-PCR) analysis

Total RNA was extracted from samples of fruits, flowers, leaves and stems of the two luffa varieties using RNA simple total RNA kit (Tiangen Biotech Beijing Co.,

Ltd., Beijing, China). The extracted RNA was reverse transcribed into cDNA and passed through EasyScript^R^ superMix (Transgen Biotech, Beijing, China) according to the manufacturer’s instructions. q-PCR was performed on an SYBR Green system (TaKaRa, Dalian, China). The cycling conditions were 3 min of predenaturation at 95°C, then 95°C for 10 s, 60°C for 30 s, and 72°C for 90 s (40 cycles). To ensure accurate normalization in our q-PCR analysis, we selected Maker00006543 (40S ribosomal protein SA-like) as the reference gene based on its stable and relatively high expression across all samples in our transcriptome dataset. While commonly used reference genes (Chen et al., 2021) failed to exhibit stable and reliable amplification in our experimental system, Maker00006543 demonstrated minimal variation in expression and consistent amplification. The FPKM values across samples are provided in **Supplementary Table 1**. Primers were designed using Primer Premier 6.0 (Premier Biosoft, International, Palo Alto, California, USA) and are listed in **Supplementary Table 2**. Three biological and technical repeats were performed. q-PCR results were analyzed using the 2^−ΔΔCt method (Kim et al., 2021) and statistical significance was determined using Student’s t-test.

## 3 Results

### 3.1 LQY showed higher browning susceptibility and oxidative stress than JD

To investigate differences in browning susceptibility between LQY and JD, fruit samples were cross-sectioned. After 15 min in temperature, LQY slices exhibited varying degrees of browning, while browning-tolerant JD showed no visible signs of browning (**Supplementary Figure S1A**). Moreover, LQY demonstrated notably higher PPO enzyme activity (**Supplementary Figure S1B**) and ROS levels (**Supplementary Figure S1C**) than JD across S1, S2, and S3. These results suggest that LQY may contain higher concentrations of polyphenols and flavonoids, which are more susceptible to oxidative changes.

### 3.2 Transcriptome analysis revealed distinct gene expression patterns between varieties and across developmental stages

**Figure 1:**
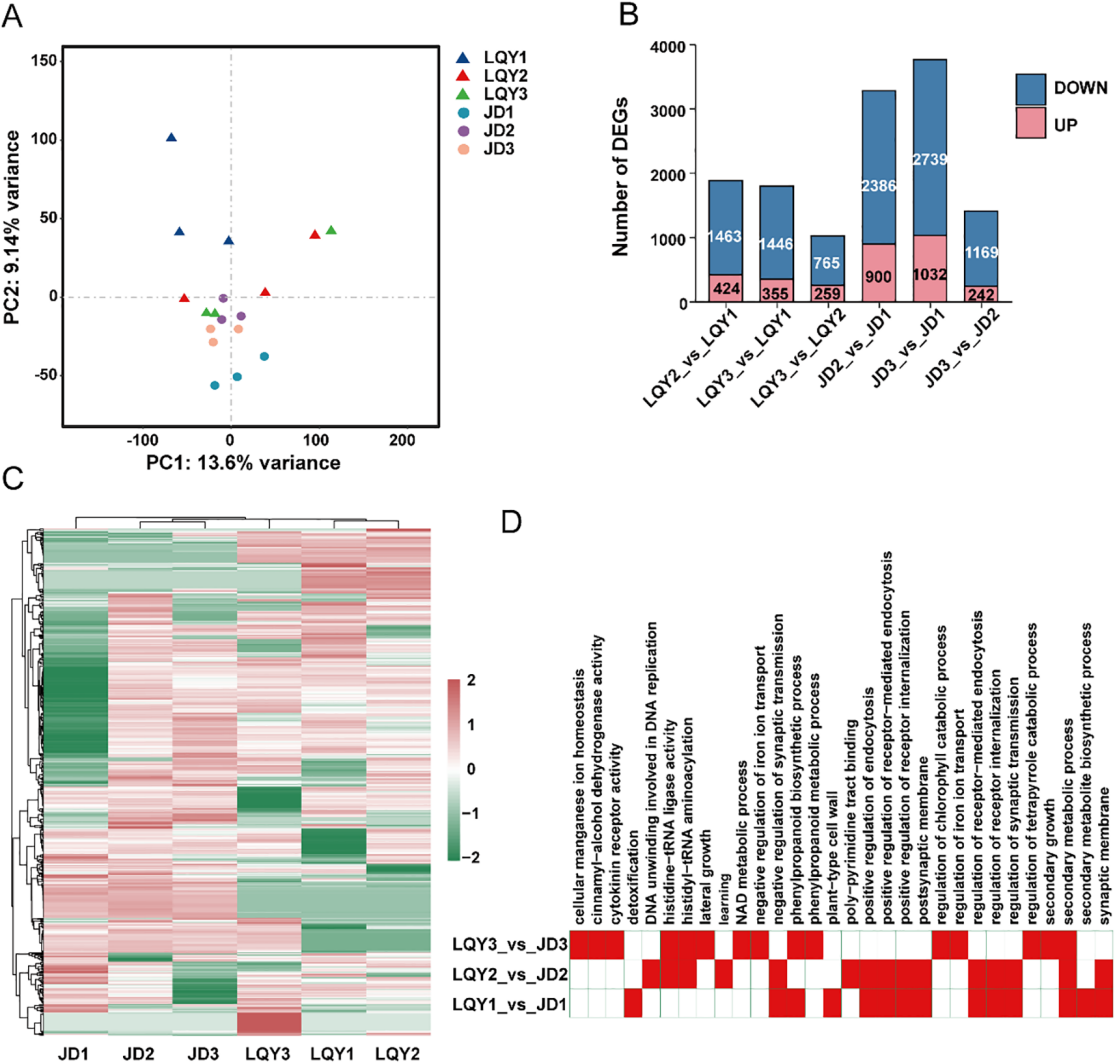
Transcriptome analysis revealed distinct patterns of gene expression between varieties and across developmental stages. **(A)** Principal component analysis (PCA) of transcriptome revealed clear clustering within each variety and distinct separation between the two varieties. **(B)** Number of differentially expressed genes (DEGs) in pairwise comparisons between stages within each variety. **(C)** Heatmap analysis of the relative expression of all DEGs revealed variety-specific differences between LQY and JD. **(D)** Functional enrichment analysis of DEGs between varieties at the same developmental stage.

PPO activity and ROS levels from earlier experiments indicated that LQY and JD may exhibit distinct polyphenol metabolism and antioxidant-related gene profiles. Therefore, a comparative transcriptome analysis was conducted at three developmental stages for both luffa varieties, with most samples generating more than 6 GB of clean data (**Supplementary Table 3**). PCA showed clear clustering within each variety as well as significant differentiation between the two varieties (**Figure 1A**). To investigate transcriptome changes across developmental stages, we performed differential expression analysis. The results revealed a higher number of DEGs in the JD variety at both S2 and S3 compared with S1, with more than double the number detected than in LQY. This may suggest more pronounced transcriptome changes in the JD variety. In addition, more DEGs were observed between S2 and S1 than between S3 and S2, implying that the most significant transcriptome changes occur during the transition from S1 to S2 (**Figure 1B**). A global expression analysis of all DEGs revealed variety-specific differences between LQY and JD (**Figure 1C**). Functional enrichment analysis of the DEGs indicated that the S1-specific DEGs were primarily associated with pathways such as phenylpropanoid biosynthesis, glutathione metabolism, oxidative phosphorylation, and plant hormone signaling pathways. At S2, DEGs were predominantly involved in beta-alanine metabolism, propanoate metabolism, and the degradation of valine, leucine, and isoleucine. At S3, the enriched pathways included phenylalanine, tyrosine, and tryptophan biosynthesis, alongside phenylpropanoid biosynthesis (**Figure 1D**). The results highlighted that both S1 and S3 exhibited active phenylalanine metabolic processes, suggesting a potential link between the phenylalanine metabolic pathway and the regulation of polyphenol and flavonoid metabolism.

### 3.3 K-means clustering of DEGs identified modules with distinct pathway enrichment and key hub genes

**Figure 2:**
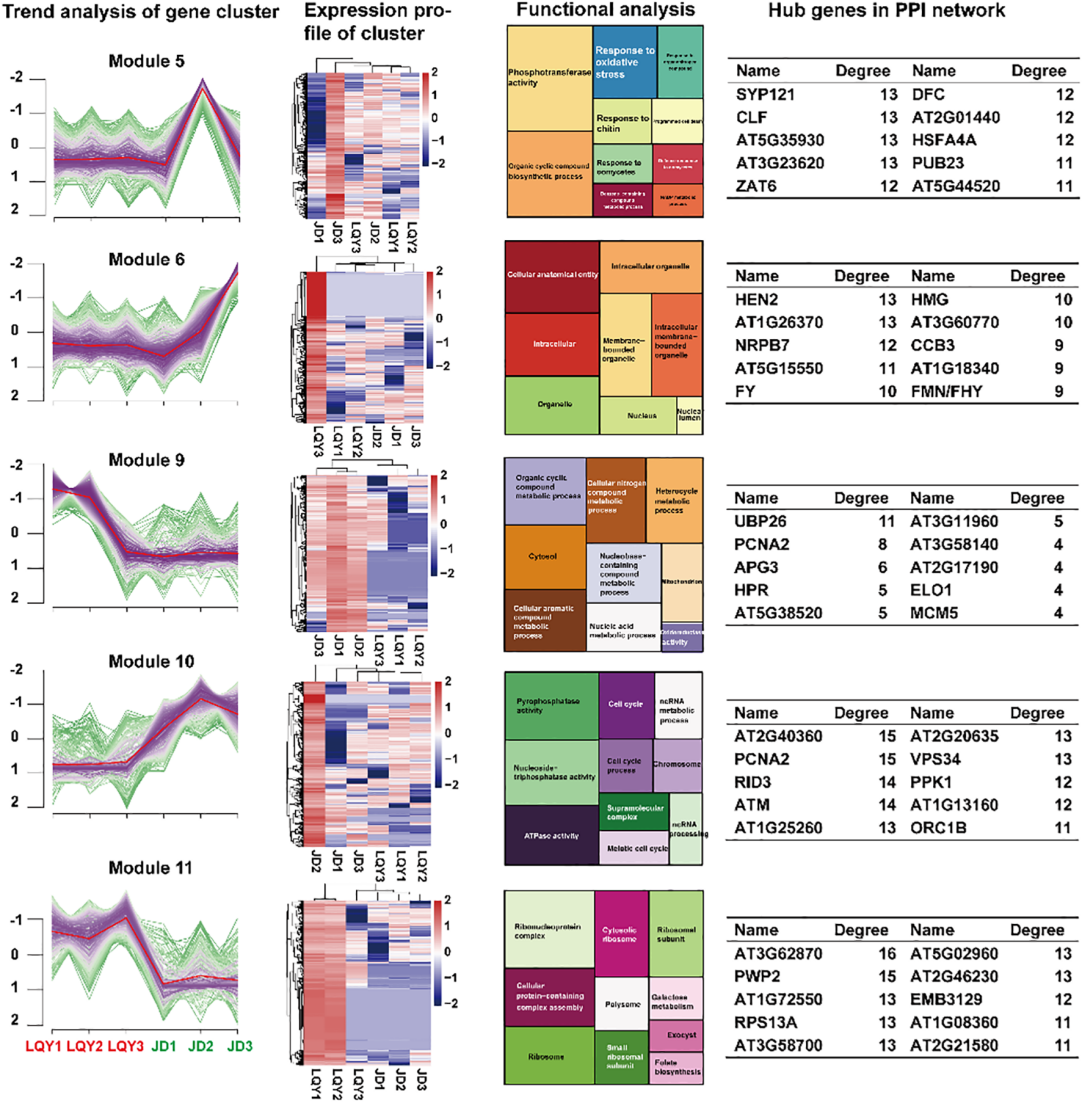
K-means cluster analysis and functional analysis of DEGs across developmental stages between LQY and JD. The first column represents the gene expression change trend of DEGs in each module, the second column presents the expression heatmap of genes in the module, the third column displays the enriched pathways of the genes in the module, and the fourth column shows the hub genes from the protein-protein interaction (PPI) network analysis of the genes in the module.

Due to the dynamic variations in the expression profiles between LQY and JD, the k-means clustering method was applied to perform cluster analysis of all DEGs (**Figure 2**). A total of 12 modules were identified, with 5 modules (Module 5, 6, 9, 10, and 11) showing opposing or divergent expression patterns between LQY and JD. Functional enrichment analysis revealed distinct characteristics among the five modules. Notably, Module 5 was upregulated in JD but not in LQY during S2, with genes in this module associated with phosphotransferase activity, response to oxidative stress, response to chitin, and response to oomycetes. This suggested that at S2, a considerable number of genes linked to stress tolerance were upregulated in JD, which may contribute to its browning tolerance properties. Protein-protein interaction (PPI) analysis of the DEGs in Module 5 revealed that *SYP121* and *CLF* may be crucial genes during the process. Furthermore, the DEGs in Module 6 were upregulated at both S2 and S3 in JD but not in LQY. Analysis of their roles suggested that these genes may be associated with cellular anatomical entities, intracellular organelles, and related processes. *HEN2*, *AT1G26370*, and *NRPB7* may play key roles in these functions. Module 9 and Module 11 revealed comparable change tendencies, with DEGs being more abundant in LQY. Functional analysis displayed that the DEGs may be associated with pathways such as oxidoreductase activity, galactose metabolism, and folate biosynthesis. *UBP26* and *AT3G11960* were identified as key genes in Module 9, while *AT3G62870* and AT5G02960 were recognized as key genes in Module 11. In Module 10, the JD variety exhibited higher expression at all stages compared with LQY, peaking at S2 before decreasing slightly. This module may be associated with pyrophosphatase activity, ATPase activity, and ncRNA processing pathways, with *AT2G40360* and *AT2G20635* as pivotal genes.

### 3.4 Metabolomic analysis revealed distinct accumulation patterns of polyphenolic and flavonoid metabolites between LQY and JD

Transcriptome analysis suggested that genes involved in polyphenolic biosynthesis may differ significantly between LQY and JD, potentially leading to variations in polyphenolic compound accumulation. Subsequent metabolomic analysis identified 13, 5, and 8 variety-specific polyphenol metabolites at S1, S2, and S3, respectively. Similarly, 18, 14, and 9 variety-specific flavonoid metabolites were detected during the same stages. In LQY, 12 out of 13 polyphenolic metabolites were upregulated at S1, while 7 out of 8 were upregulated at S3. In contrast, 13 of 18 flavonoid metabolites were upregulated in JD at S1 (**Supplementary Figure S2**). These periodic fluctuations in polyphenol and flavonoid metabolite levels indicate that LQY accumulated large amounts of polyphenolic metabolites at S1 and S3, while JD accumulated substantial amounts of flavonoid metabolites at S1.

### 3.5 Expression changes in the structural genes of the polyphenol and flavonoid metabolism pathways

**Figure 3:**
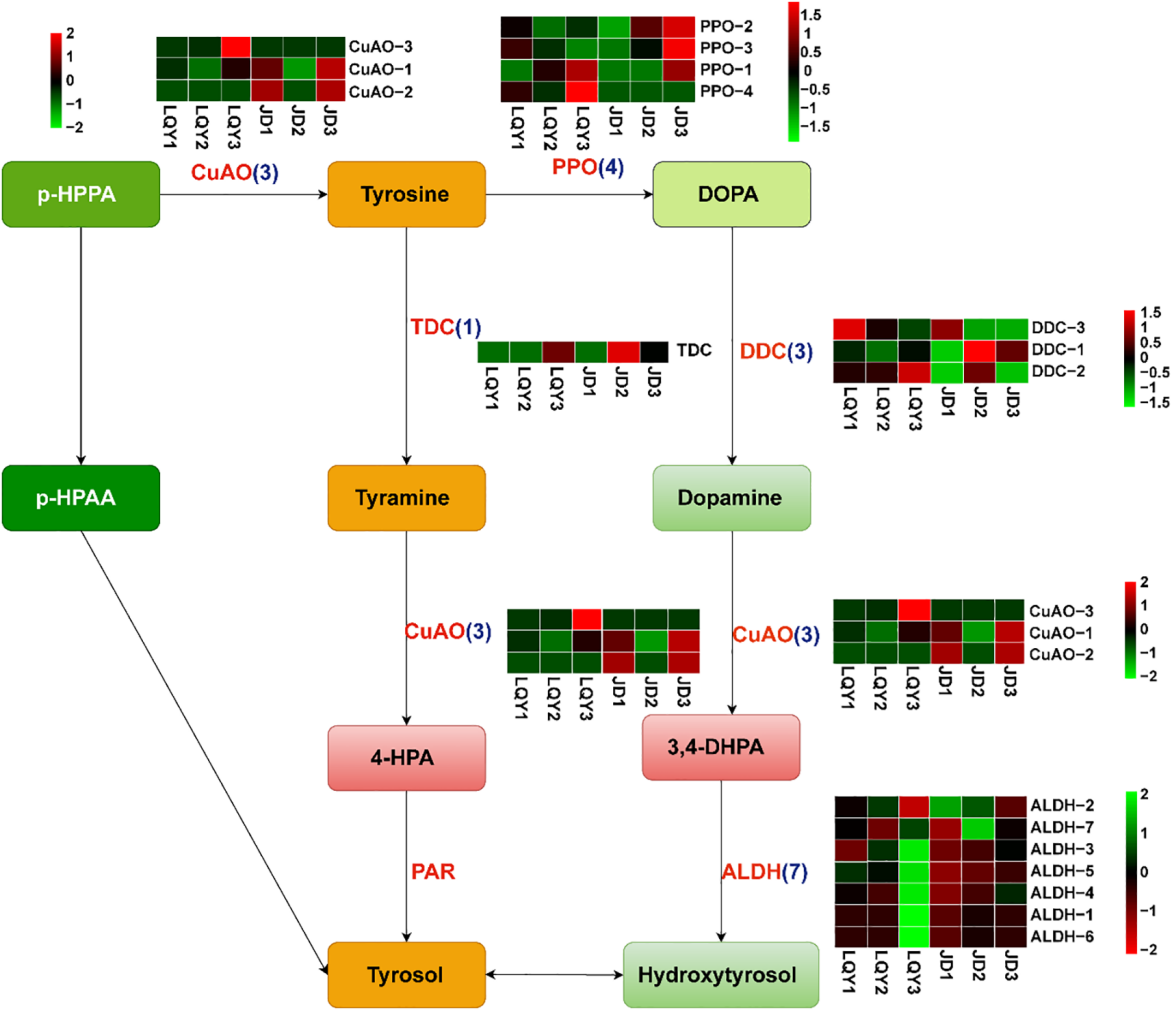
Expression changes of structural genes in polyphenol metabolic pathways at different developmental stages between LQY and JD. The heatmaps show the expression profiles of enzyme genes involved in tyrosol and hydroxytyrosol synthesis. PPO, polyphenol oxidase; DDC, DOPA decarboxylase; CuAO, copper-containing primary amine oxidase; ALDH, alcohol dehydrogenase; TDC, tyrosine decarboxylase; PAR, phenyl-acetaldehyde reductase.

To investigate the molecular mechanisms underlying secondary metabolite production, we explored expression changes in the structural genes of the polyphenol and flavonoid metabolism pathways. A total of 18 genes were identified to be involved in tyrosine and hydroxytyrosol production. *PPO* expression peaked at the S3 stage in both LQY and JD, exhibiting stage-specific regulation. Moreover, copper amine oxidase (*CuAO*) and tryptophan decarboxylase (*TDC*) showed higher expression in JD, with a distinct increase at S3. The aldehyde dehydrogenase (*ALDH*) genes, *ALDH-1*, *ALDH-3*, *ALDH-4*, *ALDH-5*, *ALDH-6*, and *ALDH-7* showed significant upregulation at S3 in LQY, potentially due to the higher polyphenol metabolic activity in this variety (**Figure 3**).

In the flavonoid metabolism pathway, we identified 57 genes from eight families, including 5 *HCT* (hydroxycinnamoyltransferase) genes, 17 *C3H* (cinnamate 3-hydroxylase) genes, 4 *CSE* (caffeoyl shikimate esterase) genes, 17 *PAL* (phenylalanine ammonia-lyase) genes, 4 *4CL* (4-coumarate-CoA ligase) genes, 4 *CHS* (chalcone synthase) genes, 4 *F3H* (flavanone 3-hydroxylase) genes, and 4 *UGT* (uridine diphosphate glycosyltransferase) genes. Further examination of structural gene expression changes in the flavonoid metabolic pathway revealed significant differences in the key genes between LQY and JD. Notably, *HCT*, *CHS*, *CSE*, and *UGT* were highly expressed in LQY at S1, while *F3H* was highly expressed in JD at S3. Other genes, such as *PAL*, *C3H*, and *4CL*, exhibited varying expression patterns between species, which may contribute to the significant accumulation of flavonoid metabolites in JD (**Supplementary Figure S3**).

### 3.6 Expression profiling of TFs involved in browning

**Figure 4:**
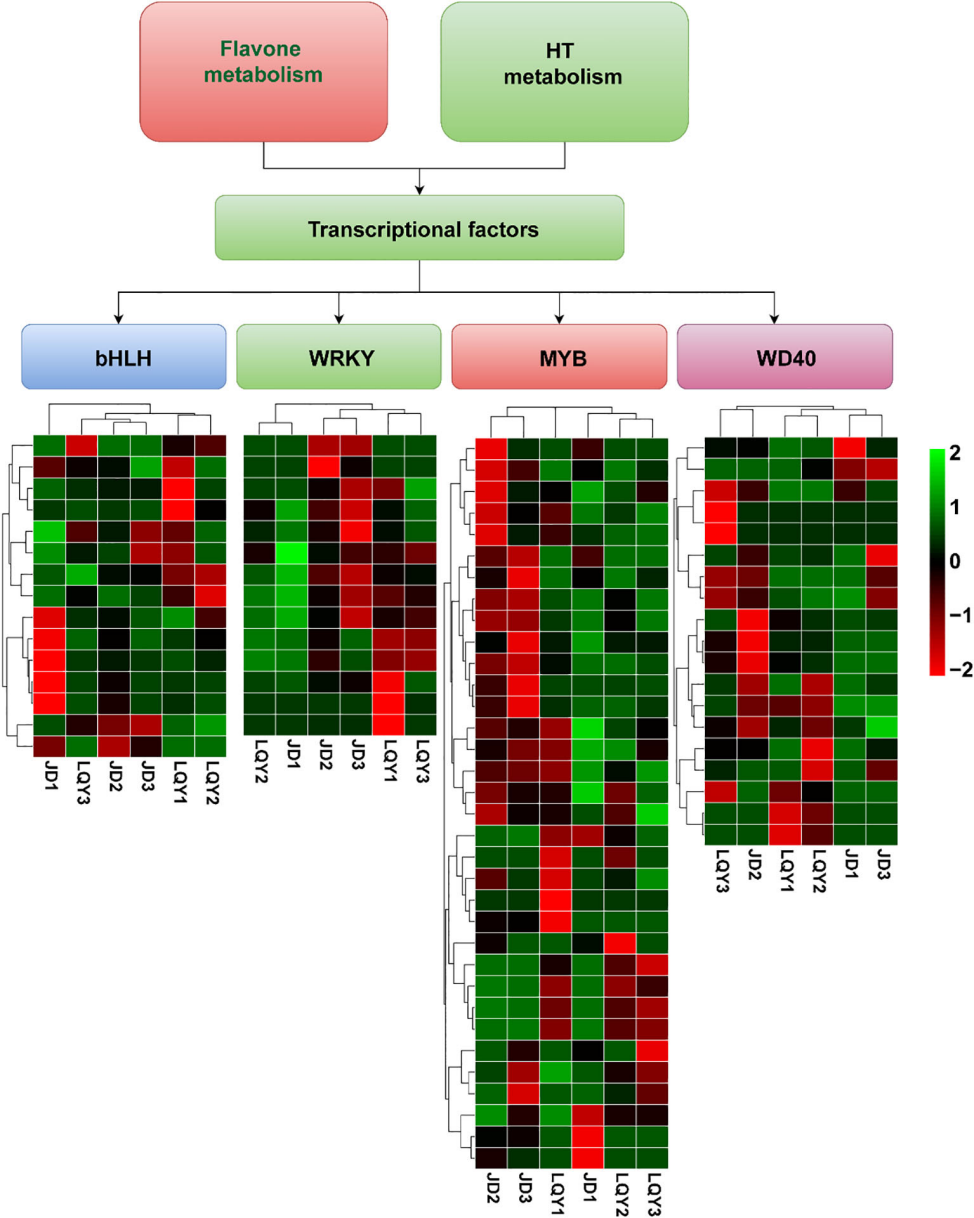
Heatmaps displaying the expression patterns of transcription factor (TF) families associated with luffa browning in LQY and JD across different developmental stages. These include 34 MYB, 15 bHLH, 19 WD40, and 14 WRKY TFs.

To identify TFs linked to polyphenol and flavonoid metabolic pathways, as well as potential pathways involved in luffa browning, we performed a transcriptome-based analysis to assess the differential expression of TFs that may regulate these processes. The results revealed differential expression of TFs between LQY and JD, including members of the MYB, WRKY, bHLH, and WD40 families. Specifically, 34 MYB, 15 bHLH, 19 WD40, and 14 WRKY TFs were identified. Notably, bHLH2–bHLH6 were highly expressed at S1 in LQY, whereas bHLH8–bHLH12 were predominantly expressed at S1 in JD. WRKY1–WRKY9 showed peak expression at S3 in JD, while WRKY6–WRKY14 were primarily expressed at S1 in the LQY variety. Furthermore, MYB1–MYB18 were strongly expressed at S2 and S3 in JD, whereas the remaining MYB TFs exhibited stage-specific expression in LQY. The WD40 TFs displayed more variable expression patterns, reflecting both variety- and stage-specific regulation (**Figure 4**).

### 3.7 Metabolomic analyses revealed variety- and stage-specific differences

To investigate the mechanisms and patterns of metabolite changes associated with luffa browning, we performed a comparative metabolomic analysis of LQY and JD fruit samples. PCA was able to distinguish the metabolic profiles of the two luffa varieties across the three developmental stages. PC1, which accounted for 72.67% of the variation, primarily separated the samples by variety. The close clustering of replicates within each group indicated strong reproducibility. Specific metabolites, such as CHCHON233 and CHCHON343, were major contributors to the observed variation, highlighting metabolic differences between the two varieties (**Supplementary Figure S4A**). Differential analysis detected 202, 84, and 150 DAMs between LYQ and JD at S1, S2, and S3, respectively. Metabolomic analysis revealed a clear distinction between LQY and JD at S1 (**Supplementary Figure S4B**), which was consistent with transcriptome profile. KEGG functional enrichment analysis of the DAMs indicated that metabolic changes at S1 were primarily associated with phenylalanine metabolism, monoterpenoid biosynthesis, flavonoid biosynthesis, tryptophan metabolism, and tyrosine metabolism (**Supplementary Figure S4C**). At S2, glycine, serine, and threonine metabolism, along with sphingolipid and flavonoid metabolism were implicated in the DAMs between varieties (**Supplementary Figure S4D**). At S3, metabolic pathways including phenylalanine metabolism and tyrosine metabolism, were highly conserved between varieties (**Supplementary Figure S4E**). Phenylalanine metabolism was highly active during at S1 and S3, aligning with DEG-enriched metabolic pathways. Metabolomic and transcriptomic evidence indicated that S1 and S3 were crucial for polyphenol production.

### 3.8 K-means clustering of DAMs identified distinct patterns of metabolic accumulation between species

**Figure 5:**
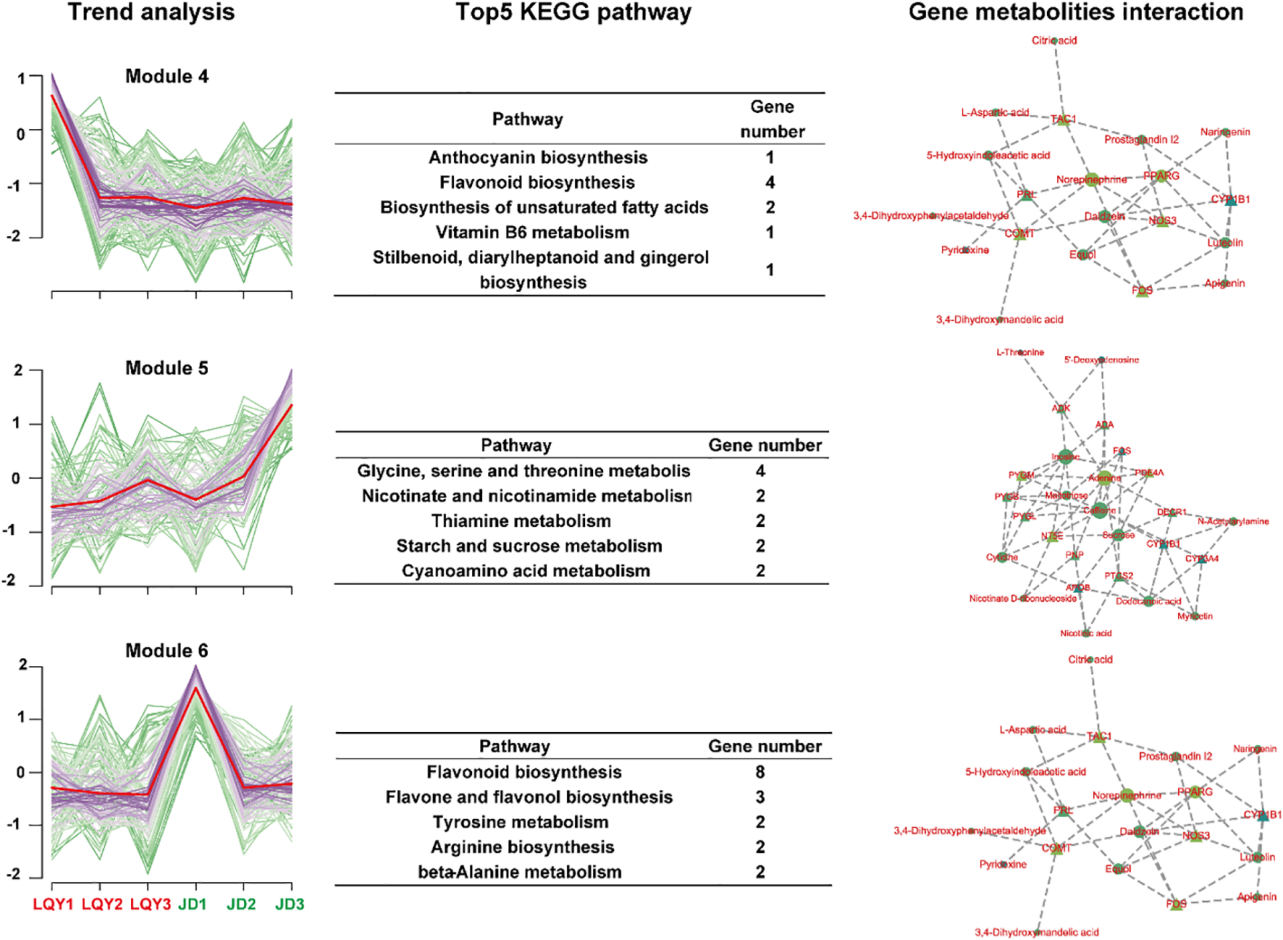
K-means clustering of DAMs identified distinct patterns of metabolic accumulation between species. The first column represents the accumulation change trend of DAMs in each module. The second column shows the top five metabolic pathways in the module. The third column displays the interaction between key metabolites and genes in each module.

We applied k-means clustering to categorize the metabolites into 12 subgroups, aiming to identify similar variation patterns within the metabolomic data. Module 4, Module 5, and Module 6 were identified as distinct metabolic modules, each exhibiting significant differences between the two varieties. In Module 4, metabolites decreased sharply at S2 in LQY, while remaining stable at all stages in JD. These metabolites were found to be associated with the biosynthesis of flavonoids, anthocyanins, and unsaturated fatty acids. Gene-metabolite interaction analysis identified *PRL*, *COMT*, and *PPARG* as key genes involved in these metabolic pathways. In Module 5, metabolites in LQY were upregulated slightly at S2 and S3, whereas in the JD variety, they exhibited notable increases. This module was primarily involved in the metabolism of glycine, serine, and threonine, as well as nicotinate, niacinamide, and thiamine. Genes such as *PTGS2*, *PYGM* and *NT5E* were identified as key genes. In contrast, Module 6 remained stable in LQY but was significantly downregulated in the JD variety at S2. This module was primarily involved in the biosynthesis pathways of flavonoids, flavones, and flavonols. *COMT*, *FOS*, and *PRL* were identified as key genes in Module 6 (**Figure 5**).

### 3.9 q-PCR analysis of *POD* and *MYB* genes

**Figure 6:**
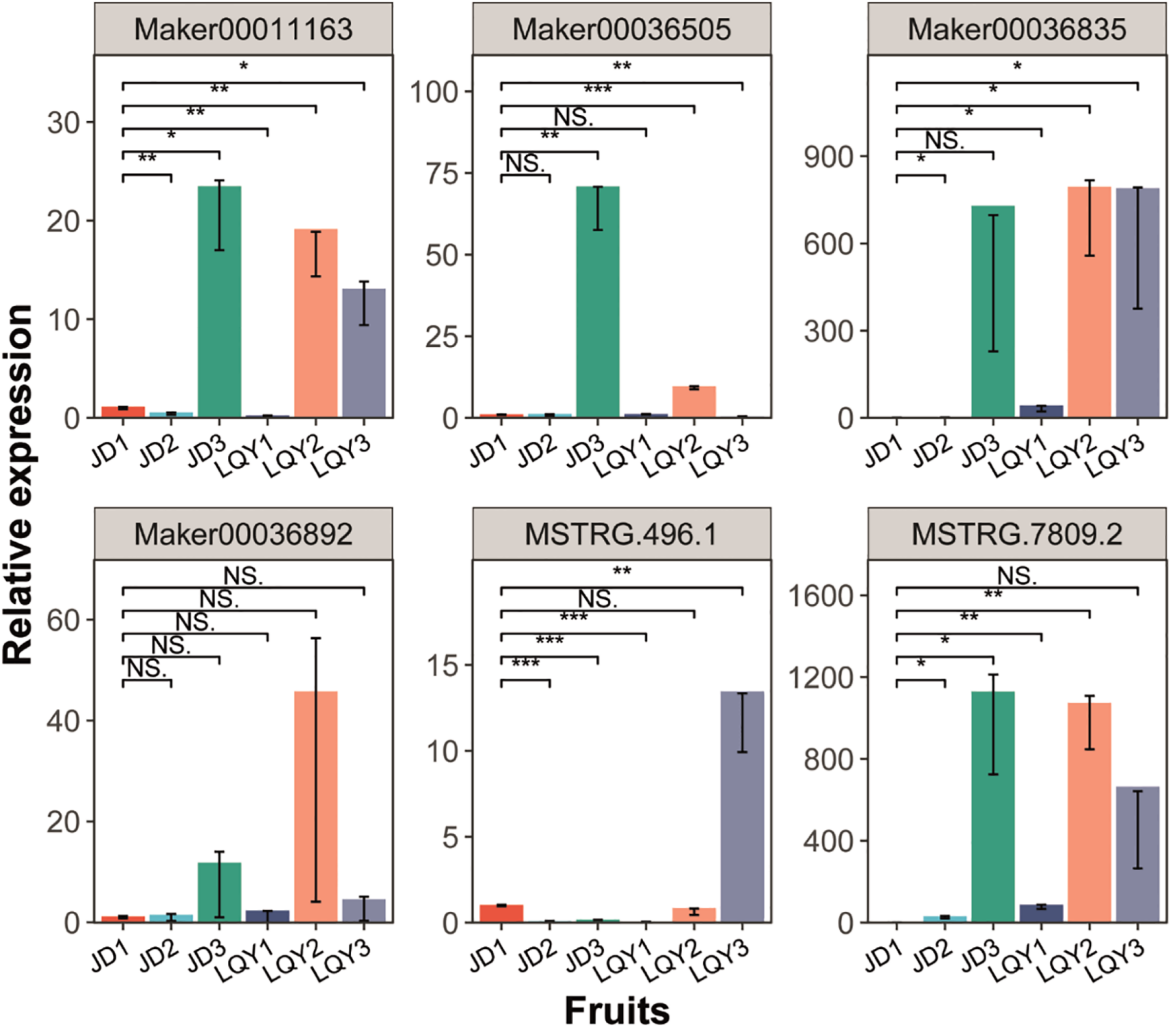
Quantitative real–time polymerase chain reaction (q-PCR) analysis of the expression of *POD* and *MYB* genes in luffa fruit samples across developmental stages. The x-axis represents different fruit samples, while the y-axis represents relative gene expression levels. Error bars indicate standard deviation. Statistical significance is denoted by asterisks (*p-value < 0.05, **p-value < 0.01, ***p-value < 0.001), while “NS” indicates non-significant differences (Student’s t-test).

The expression patterns of *POD* and *MYB* genes in the fruits (**Figure 6**), flowers (**Supplementary Figure S5A**), stems (**Supplementary Figure S5B**), and leaves (**Supplementary Figure S5C**) of LQY and JD were analyzed by q-PCR. The results indicated that the expression levels of these two gene families were highest in the fruits and lower in all other tissues. Furthermore, expression of both gene families was generally higher in LQY than in JD, with the exception of Maker00036505 in fruit. Gene expression increased with developmental stages, typically peaking at S2 in LQY, while in JD, the highest expression was observed at S3. These findings were consistent with RNA-seq results and suggest that MYB TFs and POD enzymes may regulate the expression of browning-related genes in LQY and JD, potentially contributing to browning in luffa.

## 4 Discussion

Luffa is a widely cultivated vegetable in China, with breeding efforts increasingly focused on developing high-quality, non-browning varieties. However, the molecular mechanisms governing browning-related gene expression, metabolite accumulation, and regulatory pathways remain unclear. This study employed transcriptome and metabolomic analyses to investigate the mechanisms underlying browning sensitivity and tolerance in two luffa varieties with differing browning phenotypes. Browning-sensitive LQY exhibited higher PPO activity and ROS levels compared with browning-tolerant JD. Transcriptome analysis revealed distinct gene expression patterns, with JD displaying more DEGs across developmental stages, particularly in stress tolerance pathways. K-means clustering identified key hub genes and enriched pathways related to oxidative stress and polyphenol metabolism. Metabolomic profiling showed that LQY accumulated more polyphenolic metabolites at S1 and S3, whereas JD accumulated more flavonoids at S1. Further analysis of polyphenol and flavonoid metabolism genes indicated variety- and stage-specific regulation. Additionally, MYB, WRKY, bHLH, and WD40 TF families were identified as potential regulators of browning tolerance. Metabolomic analysis highlighted distinct metabolic shifts, with phenylalanine metabolism, flavonoid biosynthesis, and monoterpenoid biosynthesis playing central roles. K-means clustering identified three key metabolic modules, revealing differences in flavonoid, amino acid, and lipid metabolism between LQY and JD. These findings suggest that transcriptional regulation and metabolic differences contribute to the distinct browning responses observed in LQY and JD.

Luffa browning is a form of enzymatic browning primarily caused by the oxidation of intracellular polyphenols into quinones, catalyzed by PPO and POD. These quinones undergo polymerization, forming black or brown precipitates. Previous studies have established a significant correlation between PPO activity and browning in various fruits and vegetables, such as apples (Yu et al., 2021), peaches (Zhang et al., 2025), potatoes (Guan et al., 2025), and lotus roots (Li et al., 2023). In this study, LQY exhibited higher PPO activity than JD, consistent with its greater browning sensitivity. In addition, ROS levels in LQY were also higher than that in JD, which were induced by mechanical damage. This ROS accumulation accelerated membrane lipid peroxidation, thereby promoting browning (Yang, 2009; Yi et al., 2010; Lin et al., 2014). These results align with previous studies, further supporting that luffa browning is primarily enzymatic, and that PPO and ROS may play a synergistic role in the browning process of fresh-cut luffa. Phenolic substances, PPO, and oxygen are the three key conditions for enzymatic browning. Phenolic substances serve as the primary substrates for PPO in this process, with their types and concentrations playing crucial roles in determining the browning characteristics of fruits and vegetables (Liu et al., 2010). Metabolomics has successfully analyzed the browning mechanisms in various fruits and vegetables (Tao et al., 2021; Wang et al., 2023; Gao et al., 2024). For example, Wang et al. found that the browning process in apple peel was primarily influenced by the accumulation of phenolic compounds and flavonoids, as revealed by transcriptome and metabolomics analyses (Wang et al., 2023). In our study, a total of 26 phenolic acids were identified as DAMs between LQY and JD. Among them, gentisic acid, salidroside, myzodendrone, phloretic acid, protocatechuic acid and salicylic acid were more abundant in LQY than in JD. In a previous study, gentisic acid and myzodendrone were identified as the main phenolic acids in luffa (Wang et al., 2021). Zhu et al. reported changes in phenolic acid levels, including an increase in gentisic acid, in browned luffa slices (Zhu et al., 2017). In our study, we detected a higher accumulation of gentisic acid in the browning-sensitive variety LQY compared to the browning-tolerant variety JD. This finding further supports the potential role of gentisic acid in enzymatic browning.

Among the identified DAMs, in addition to phenolic acids involved in the phenylpropanoid metabolic pathway, flavonoids and their derivatives also exhibited significant differences between LQY and JD. Specifically, the accumulation of (−)-maackiain, 2-hydroxydaidzein, cianidanol, apigenin, and cynaroside was higher in JD than in LQY, consistent with the findings of Wang et al. (2021). These results provide further evidence that flavonoids contribute to the formation of browning-related products. Numerous studies have demonstrated that the upregulation of genes involved in the oxidation of phenolic compounds is a key indicator of browning in fruits and vegetables. Pang et al. found that exogenous selenium treatment significantly reduced the expression of PPO- and POD-related genes in apples, leading to decreased phenolase activity and reduced membrane lipid peroxidation, thereby exerting an anti-browning effect (Gao et al., 2024). Similarly, suppression of PPO gene expression in potatoes resulted in reduced tuber browning (Chi et al., 2014). In transgenic sugarcane, PPO overexpression resulted in higher PPO activity and severe browning of sugarcane juice, highlighting a strong correlation between PPO gene expression and browning (Vickers et al., 2005). Give this strong correlation, this study primarily focused on the oxidase genes involved in browning process (Vaughn and Duke, 1984; Tang et al., 2020). In the biosynthesis pathway of phenolic substances, we observed that *PPO-2*, *PPO-3* and *PPO-4* were highly expressed in LQY, but showed significantly lower expression in JD at S1. This result was consistent with the findings of Chen et al., who reported that the expression of *POD* genes were downregulated in the browning-tolerant luffa variety YLB05 (Chen et al., 2015). In addition, the genes encoding key enzymes involved in flavonoid metabolism, such as *PAL*, *C3H*, and *4CL*, showed different expression patterns between LQY and JD. Phenylalanine ammonia-Lyase (PAL; EC 4.3.1.5) is a key enzyme in the biosynthesis of phenolic compounds, and the increase of PAL activity promotes enzymatic browning in fruits and vegetables (Zhao et al., 2024). The PAL gene was found to be upregulated in the high-browning cultivar 'Weiningdahuangli' compared with the low-browning cultivar ‘Eli No.2’. (Fan et al., 2021). Similarly, another research on the explants of *Phalaenopsis* explants further confirmed the regulatory role of the *PAL* gene in browning (Xu et al., 2007). Additionally, four *PAL* genes were identified to be highly expressed in the browning-sensitive luffa cultivar XTR05 (Chen et al., 2015). Based on our research results, POD and PAL are key enzymes involved in enzymatic browning, and the genes regulating these enzymes may play a crucial role in the browning process of luffa.

WRKY TFs are key regulators of gene expression in the reactive oxygen pathway, playing essential roles in plant responses to biotic, abiotic, and oxidative stress (Eulgem and Somssich, 2007; Dan et al., 2016). Previous studies have shown that WRKY and MYB TFs function similarly to PPO in regulating apple browning (Xinyue et al., 2023). In luffa, Zhu et al. suggested that members of the group II WRKY superfamily may be associated with browning (Zhu et al., 2017). Additionally, Liu et al. found that four *WRKY* genes were upregulated during different stages of postharvest and storage browning, indicating their potential regulatory roles in this process (Liu et al., 2017). Wang et al. also reported that WRKY members may be involved in luffa browning regulation (Wang et al., 2020). In this study, 34 MYB and 14 WRKY TFs were identified, which exhibited variety-specific expression patterns. This potential association between these TFs and browning in Luffa warrants further investigation. Structural and functional analyses are required to determine whether the identified candidate genes contribute to browning in LQY and JD.

## 5 Conclusions

**Figure 7:**
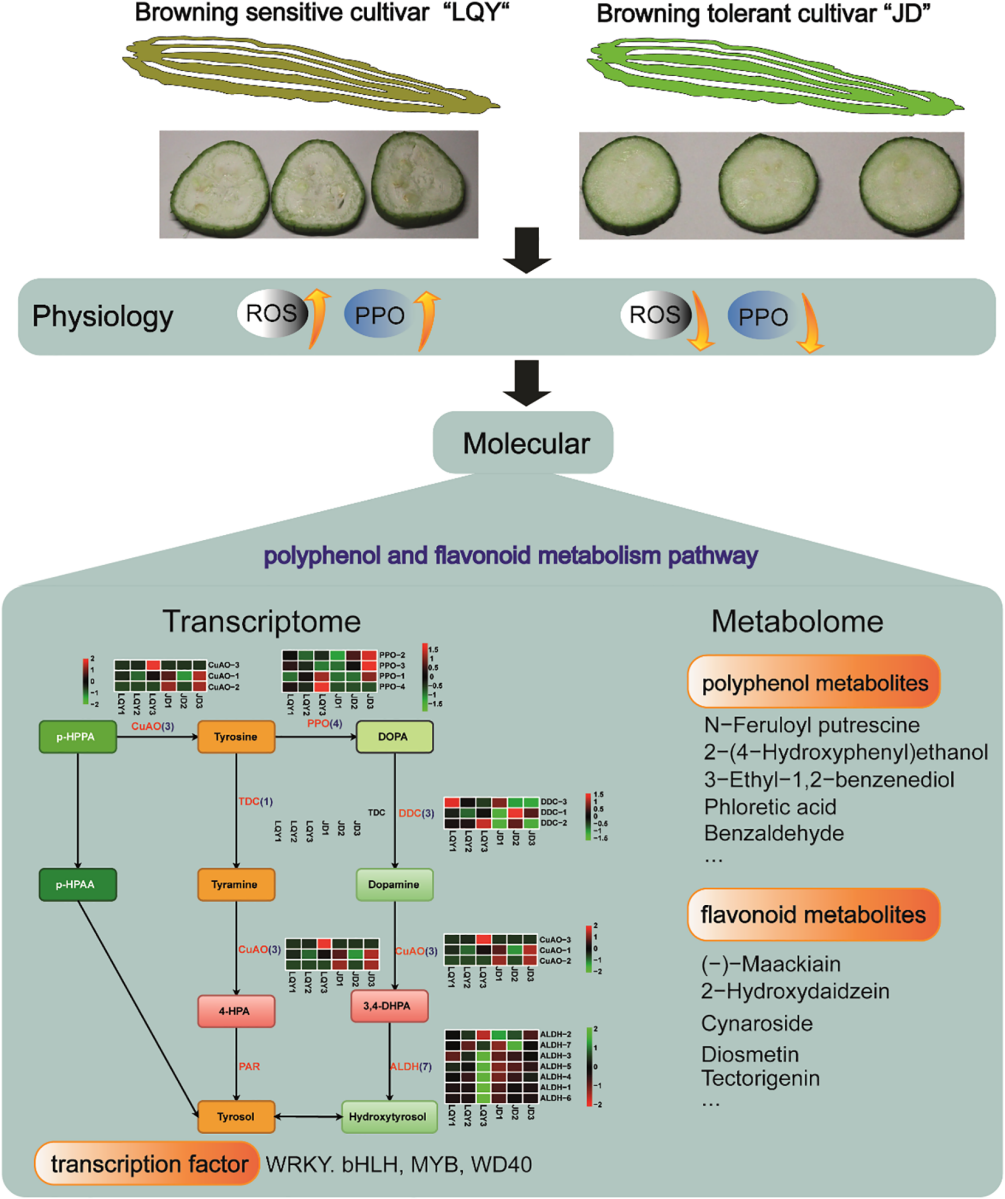
Schematic diagram demonstrating the physiological and molecular differences between browning-sensitive LQY and browning-tolerant JD luffa varieties.

In this study, transcriptome and metabolomics analyses were integrated to investigate the molecular regulation of browning in two luffa varieties, browning-sensitive LQY and browning-tolerant JD. DEGs and DAMs involved in polyphenol and flavonoid metabolism pathways were identified. In addition, WRKY, bHLH, MYB and WD40 TFs exhibited differential expression between the two luffa varieties, suggesting their potential roles in browning regulation (**Figure 7**). q-PCR analysis confirmed the expression levels of *POD* and *MYB* genes in luffa tissues. Except for Maker00036505, the expression levels of other genes in LQY were higher than those in JD. This study enhances our understanding of the molecular mechanisms of luffa browning, and the identified candidate genes provide valuable resources for future research on breeding of browning-tolerant varieties.

## Data Availability

The datasets presented in this study can be found in online repositories. The names of the repository/repositories and accession number(s) can be found below: https://www.ncbi.nlm.nih.gov/, PRJNA935857.

## Supplementary material

Supplementary Figure 1 The Long-quan-yi (LQY) variety showed higher browning susceptibility and oxidative stress than the Jiang-du (JD) variety. **(A)**: Cross-sectional comparison of luffa slices between LQY and JD varieties. **(B)**: Polyphenol oxidase (PPO) activity in LQY and JD at 15 (S1), 20 (S2), and 45 days (S3) following flowering. **(C)**: Reactive oxygen species (ROS) levels in LQY and JD across the three developmental stage.

Supplementary Figure 2 Heatmap demonstrating the accumulation patterns of polyphenol and flavonoid metabolites at S1, S2 and S3 in LQY and JD samples.

Supplementary Figure 3 Expression changes of structural genes in flavonoid metabolic pathways at different developmental stages between LQY and JD. PAL, phenylalanine ammonia lyase; 4CL, 4-coumarate CoA ligase; HCT, p-hydroxycinnamoyl CoA quinate shikimate p-hydroxycinnamoyl transferase; C3H, p-coumarate 3-hydroxylase; CSE, caffeoyl shikimate esterase; COMT, caffeic acid O-methyl transferase; CHS, chalcone synthase; CHI, chalcone isomerase; UGT, UDP-glucosyltransferases; F3H, flavanone 3-hydroxylase.

Supplementary Figure 4 Metabolomic analyses revealed variety- and stage-specific differences. **(A)**: PCA plot showing the separation of different luffa varieties at various stages. Metabolites in the plot, such as CHCHON343, may be major contributors to the observed variation. **(B)**: Venn diagram illustrating the overlap of DEGs between species at the same stage. **(C–E)**: Treemaps visualizing the metabolic pathways associated with DEGs between the two luffa varieties at S1 **(C)**, S2 **(D)**, and S3 **(E)**.

Supplementary Figure 5 q-PCR analysis of the expression of *POD* and *MYB* genes in luffa flower, stem and leaf samples across developmental stages. The x-axis represents different samples, while the y-axis represents relative gene expression levels. Error bars indicate standard deviation. Statistical significance is denoted by asterisks (*p-value < 0.05, **p-value < 0.01, ***p-value < 0.001), while “NS” indicates non-significant differences.
