# Supplementary figures and images for: Insights into the molecular mechanisms of browning tolerance in luffa: a transcriptome and metabolome analysis
Source: Front Plant Sci. 2025 Jun 10;16:1530531. doi: 10.3389/fpls.2025.1530531 (PMC12186849; doi:10.3389/fpls.2025.1530531)

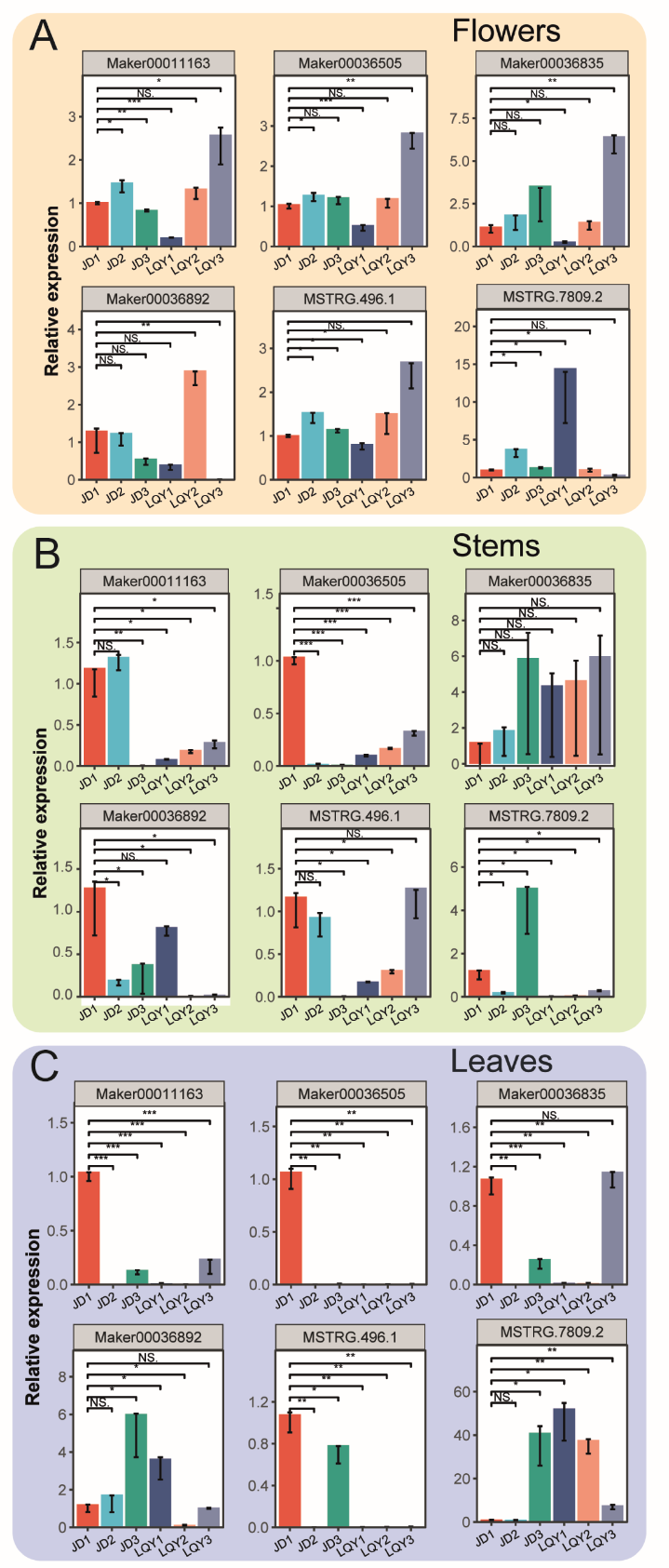

Supplement: Supplementary Figure 1 — The Long-quan-yi (LQY) variety showed higher browning susceptibility and oxidative stress than the Jiang-du (JD) variety. (A): Cross-sectional comparison of luffa slices between LQY and JD varieties. (B): Polyphenol oxidase (PPO) activity in LQY and JD at 15 (S1), 20 (S2), and 45 days (S3) following flowering. (C): Reactive oxygen species (ROS) levels in LQY and JD across the three developmental stage. [file Image1.tif]

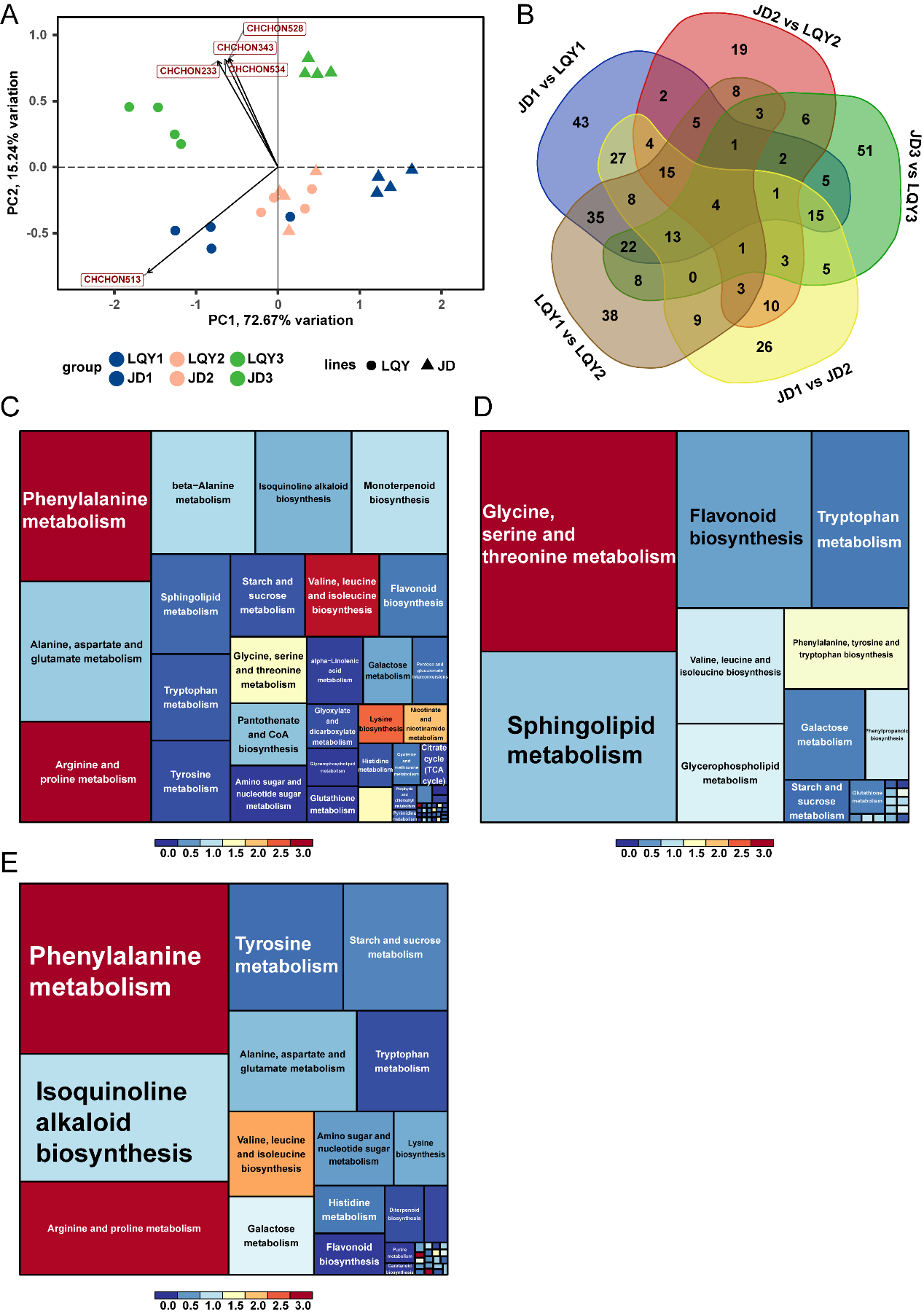

Supplement: Supplementary Figure 2 — Heatmap demonstrating the accumulation patterns of polyphenol and flavonoid metabolites at S1, S2 and S3 in LQY and JD samples. [file Image2.tif]

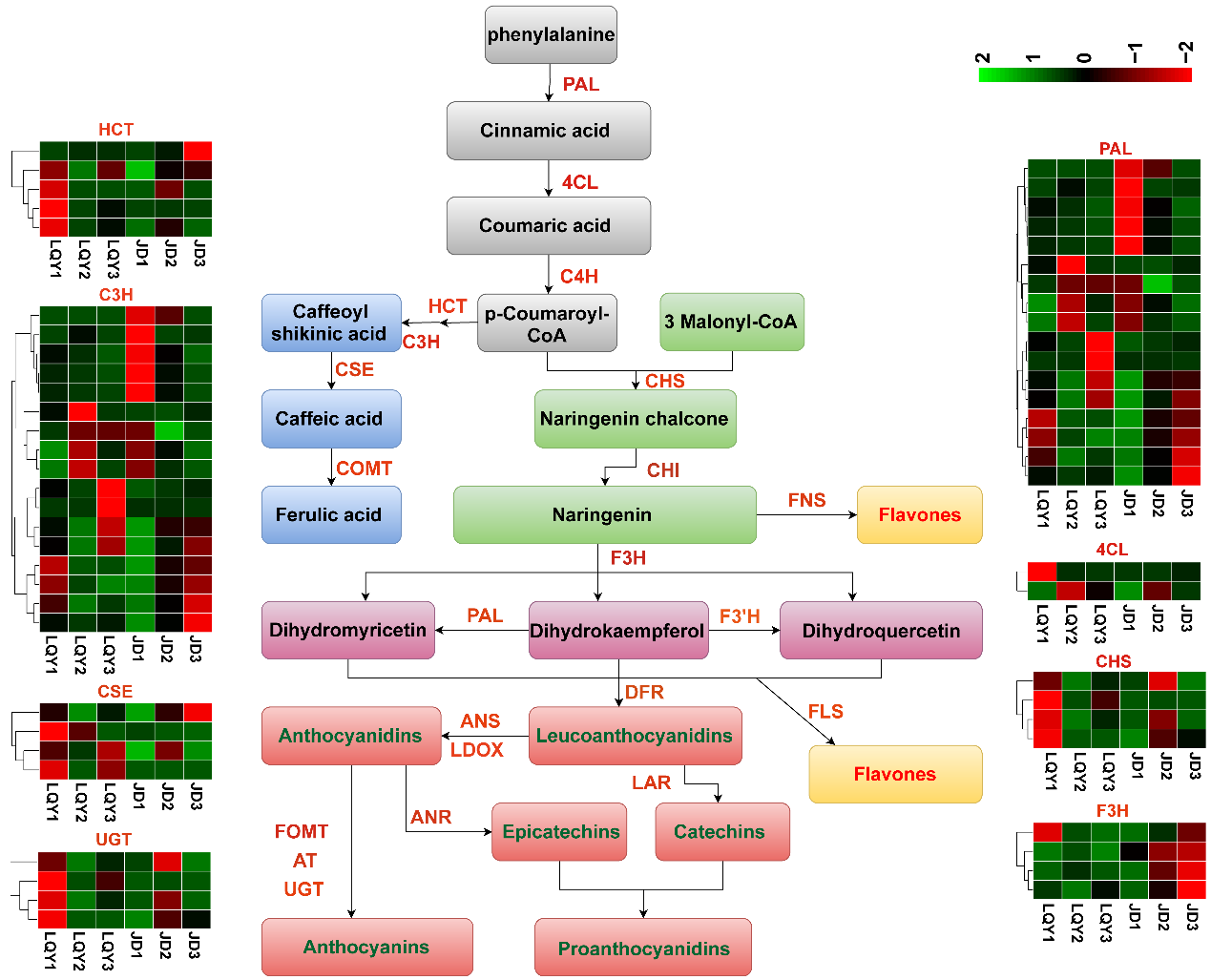

Supplement: Supplementary Figure 3 — Expression changes of structural genes in flavonoid metabolic pathways at different developmental stages between LQY and JD. PAL, phenylalanine ammonia lyase; 4CL, 4-coumarate CoA ligase; HCT, p-hydroxycinnamoyl CoA quinate shikimate p-hydroxycinnamoyl transferase; C3H, p-coumarate 3-hydroxylase; CSE, caffeoyl shikimate esterase; COMT, caffeic acid O-methyl transferase; CHS, chalcone synthase; CHI, chalcone isomerase; UGT, UDP-glucosyltransferases; F3H, flavanone 3-hydroxylase. [file Image3.tif]

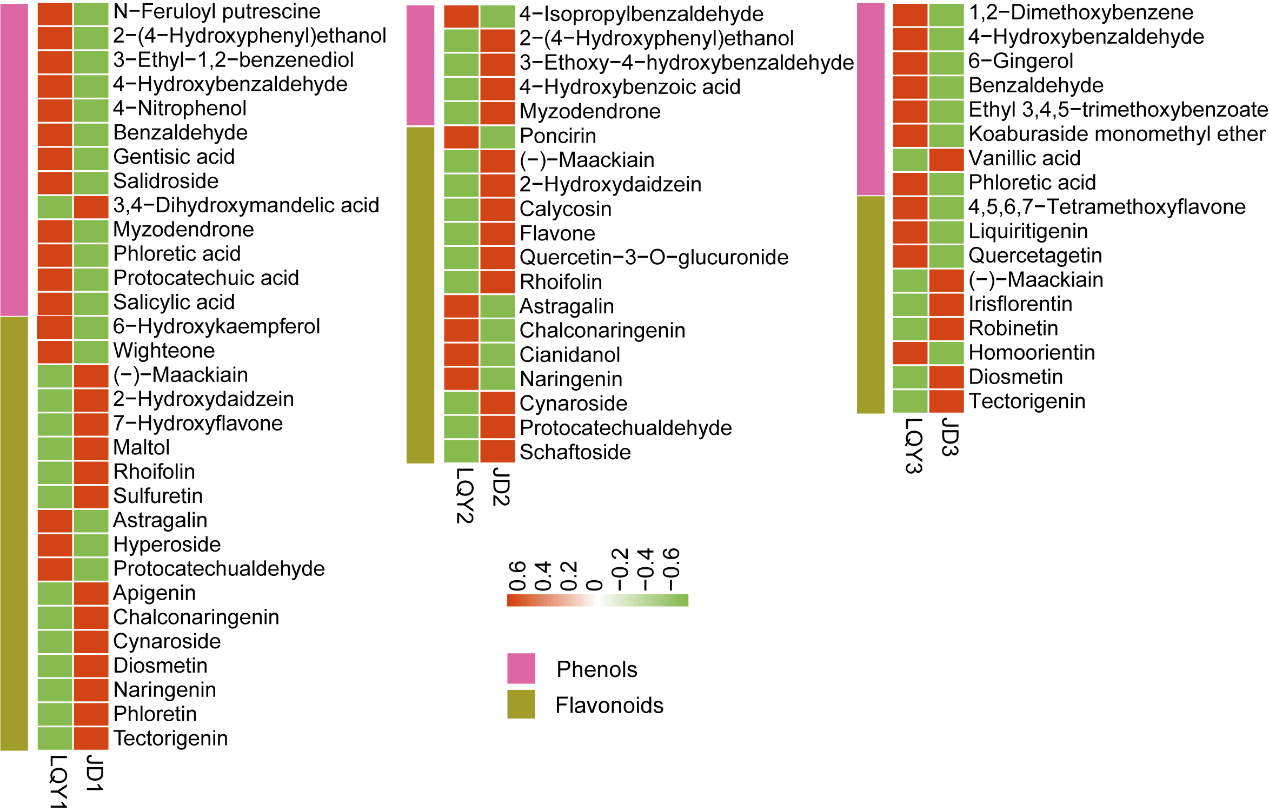

Supplement: Supplementary Figure 4 — Metabolomic analyses revealed variety- and stage-specific differences. (A): PCA plot showing the separation of different luffa varieties at various stages. Metabolites in the plot, such as CHCHON343, may be major contributors to the observed variation. (B): Venn diagram illustrating the overlap of DEGs between species at the same stage. (C–E): Treemaps visualizing the metabolic pathways associated with DEGs between the two luffa varieties at S1 (C), S2 (D), and S3 (E). [file Image4.tif]

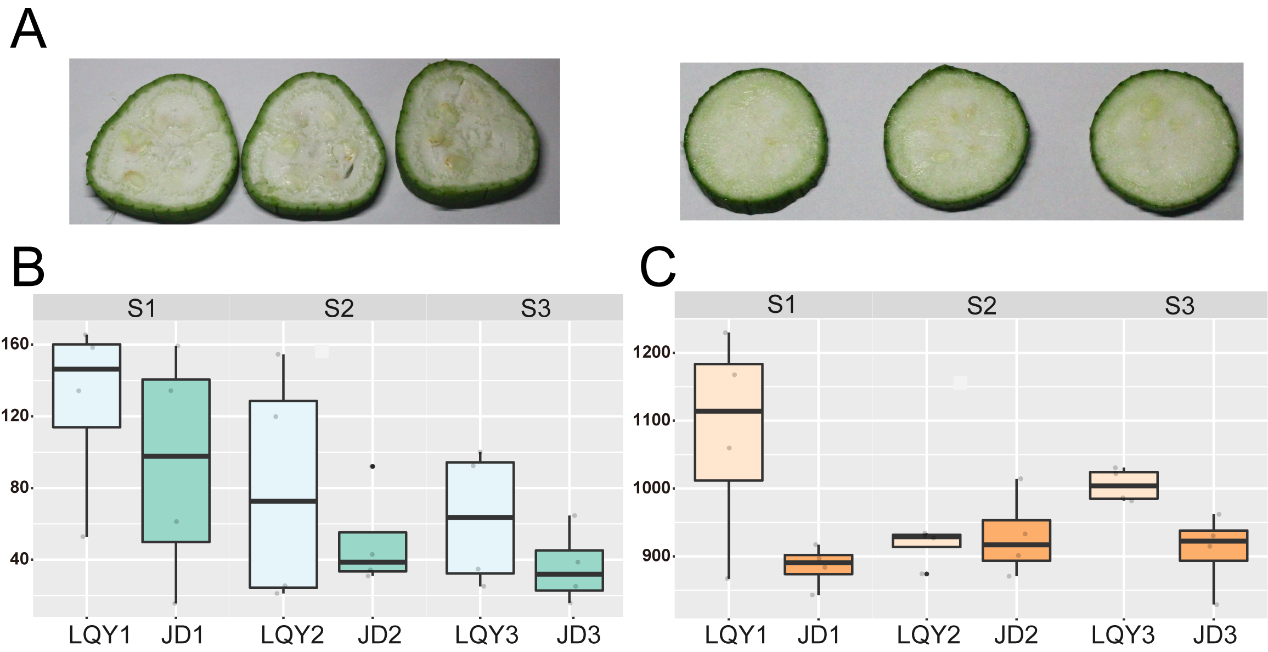

Supplement: Supplementary Figure 5 — q-PCR analysis of the expression of POD and MYB genes in luffa flower, stem and leaf samples across developmental stages. The x-axis represents different samples, while the y-axis represents relative gene expression levels. Error bars indicate standard deviation. Statistical significance is denoted by asterisks (*p-value < 0.05, **p-value < 0.01, ***p-value < 0.001), while “NS” indicates non-significant differences. [file Image5.tif]
