# Supplementary material for: Insights into the molecular mechanisms of browning tolerance in luffa: a transcriptome and metabolome analysis
Source: Front Plant Sci. 2025 Jun 10;16:1530531. doi: 10.3389/fpls.2025.1530531 (PMC12186849; doi:10.3389/fpls.2025.1530531)
Supplement: Supplementary file 6 [file Table1.docx]

**Table S1 FPKM values of the q-PCR reference gene (Maker00006543) in transcriptomic data**

| Sample | FPKM value |
| --- | --- |
| BS2-2 | 143.509933 |
| ES3-2 | 121.875511 |
| BS1-3 | 167.06308 |
| ES3-3 | 128.23877 |
| ES3-1 | 129.449997 |
| BS1-2 | 190.863647 |
| ES1-3 | 172.933823 |
| ES1-1 | 173.168488 |
| ES2-3 | 134.368576 |
| ES1-2 | 197.966797 |
| ES2-2 | 134.964279 |
| BS3-1 | 131.811737 |
| BS1-1 | 155.866821 |
| BS2-1 | 151.984848 |
| BS3-3 | 137.186356 |
| ES2-1 | 148.679581 |
| BS2-3 | 160.569229 |
| BS3-2 | 111.408524 |
