# Supplementary material for: Insights into the molecular mechanisms of browning tolerance in luffa: a transcriptome and metabolome analysis
Source: Front Plant Sci. 2025 Jun 10;16:1530531. doi: 10.3389/fpls.2025.1530531 (PMC12186849; doi:10.3389/fpls.2025.1530531)
Supplement: Supplementary file 7 [file Table2.docx]

**Table S2 Primer information**

| **Primer** | **F Primer Sequence (5' to 3')** | **Tm Value (°C)** | **R Primer Sequence (5' to 3')** | **Tm Value (°C)** |
| --- | --- | --- | --- | --- |
| MSTRG.496.1 (MYB) | ATGATAATGCCTTGAATGTTGTGTT | 60.0 | TTGCACTTCCATTTGCGCTC | 62.1 |
| MSTRG.7809.2 (MYB) | CTTGGCCATCGCTCATAGGT | 60.1 | GGCGACTCCATTGGCTAAGA | 60.2 |
| Maker00011163 (MYB) | AGCCAAAGGGGGAATCAAGG | 62.8 | ACAGAGTTGGAAGGGGGAGT | 57.3 |
| Maker00036835 (POD) | CGATGTGTTCCGCGATCAAC | 61.9 | GTTCAGCACACTTGGCTTGG | 58.8 |
| Maker00036892 (POD) | CGATCCGAAGGGCATGAGAA | 63.1 | TGTTTTCCACAGTTCCCGCT | 60.4 |
| Maker00036505 (POD) | GAGCGAATCTGTGCCCATCT | 59.8 | AGCGGTGAATCTGGGTTGTT | 59.3 |
| Maker00006543 (40S ribosomal protein SA-like) | CAAGGGGAAACATAGCATAGGG | 60.6 | CAAAATCTGGGGGCGGAAG | 62.6 |
