# Supplementary material for: Insights into the molecular mechanisms of browning tolerance in luffa: a transcriptome and metabolome analysis
Source: Front Plant Sci. 2025 Jun 10;16:1530531. doi: 10.3389/fpls.2025.1530531 (PMC12186849; doi:10.3389/fpls.2025.1530531)
Supplement: Supplementary file 8 [file Table3.docx]

**Table S3 Quality metrics of transcriptome data**

| sample_name | total_reads | total_bases | Q20_bases | Q30_bases | gc_content |
| --- | --- | --- | --- | --- | --- |
| JD_S1-1 | 40638338 | 6093377594 | 5976290543 | 5746221309 | 0.44569 |
| JD_S1-2 | 41138294 | 6168161364 | 6050404735 | 5816785179 | 0.445401 |
| JD_S1-3 | 44290122 | 6639131488 | 6500915503 | 6237316302 | 0.449312 |
| JD_S2-1 | 44939748 | 6738799656 | 6608704942 | 6348278461 | 0.440915 |
| JD_S2-2 | 40836278 | 6123440890 | 6002289661 | 5764051229 | 0.445458 |
| JD_S2-3 | 39338090 | 5898515402 | 5760881707 | 5497169797 | 0.441226 |
| JD_S3-1 | 40732024 | 6107726718 | 5981759860 | 5739266443 | 0.440461 |
| JD_S3-2 | 37546336 | 5629797696 | 5526709051 | 5320360524 | 0.440515 |
| JD_S3-3 | 39286614 | 5890986174 | 5775025944 | 5545824874 | 0.444401 |
| LQY_S1-1 | 41236118 | 6183320838 | 6058444664 | 5815992507 | 0.433572 |
| LQY_S1-2 | 40141788 | 6019141912 | 5904117062 | 5674321620 | 0.439761 |
| LQY_S1-3 | 42031272 | 6302110108 | 6176912956 | 5931429309 | 0.441669 |
| LQY_S2-1 | 39294616 | 5890732144 | 5751921909 | 5484628219 | 0.448107 |
| LQY_S2-2 | 37291310 | 5591550778 | 5484603892 | 5271208329 | 0.445359 |
| LQY_S2-3 | 44759742 | 6710500990 | 6544434486 | 6240403377 | 0.442901 |
| LQY_S3-1 | 44800480 | 6717776182 | 6588845319 | 6334059013 | 0.445255 |
| LQY_S3-2 | 45356392 | 6801135916 | 6664966779 | 6396373806 | 0.439289 |
| LQY_S3-3 | 41558390 | 6229116854 | 6107831089 | 5865853742 | 0.448554 |
